# Rare Chromosomal Uniformity in Black Flies of the *Simulium striatum* Species Group (Diptera: Simuliidae)

**DOI:** 10.3390/insects16050511

**Published:** 2025-05-10

**Authors:** Peter H. Adler, Sergey Vlasov, Yao-Te Huang, Upik K. Hadi, Khamla Inkhavilay, Banchai Malavong, Varvara Topolenko, Bhuvadol Gomontean, Waraporn Jumpato, Ronnalit Mintara, San Namtaku, Isara Thanee, Wannachai Wannasingha, Komgrit Wongpakam, Chavanut Jaroenchaiwattanachote, Pairot Pramual

**Affiliations:** 1 Department of Plant and Environmental Sciences, Clemson University, Clemson, SC 29634, USA; padler@clemson.edu; 2 Department of General Biology and Bioecology, Federal State University of Education, Moscow 105005, Russia; svvlasov54@gmail.com (S.V.); topolenko25@gmail.com (V.T.); 3 R & D Center Composites Department, Nan Ya Plastics Corporation, New Taipei City 238032, Taiwan; cckaky@hotmail.com; 4 Division of Medical Entomology and Parasitology, School of Veterinary Medicine and Biomedical Sciences, IPB University, Bogor 16680, Indonesia; upikke@apps.ipb.ac.id; 5 Center of Excellence in Biodiversity, National University of Laos, Vientiane 7322, Laos; khamla.inkhavilay@nuol.edu.la (K.I.); banchaimlv@gmail.com (B.M.); 6 Department of Biology, Faculty of Science, Mahasarakham University, Mahasarakham 44150, Thailand; bhuvadol.g@msu.ac.th (B.G.); waraporn.a2536@gmail.com (W.J.); ronnalitmintara@gmail.com (R.M.); isara.th@msu.ac.th (I.T.); 7 Department of Science and Mathematics, Faculty of Science and Health Technology, Kalasin University, Kalasin 46230, Thailand; san.na@ksu.ac.th; 8 Center of Excellence in Biodiversity Research, Mahasarakham University, Mahasarakham 44150, Thailand; wannachai.w@msu.ac.th (W.W.); chavanut.j@msu.ac.th (C.J.); 9 Walai Rukhavej Botanical Research Institute, Mahasarakham University, Mahasarakham 44150, Thailand; komwongpa@gmail.com

**Keywords:** aquatic insects, biodiversity, cryptic species, homosequential species, taxonomy

## Abstract

We conducted a comparative chromosomal analysis of 10 nominal species and 3 unidentified species in the *Simulium striatum* group from six countries. A total of 66 chromosomal rearrangements were found, of which 78.8% were inversions. The group is defined by 11 fixed inversions, of which 6 are unique, supporting the monophyletic status previously indicated by morphological and molecular characters. Only 1 of the 13 taxa had a unique fixed chromosomal rearrangement. Although the group demonstrates significant macrogenomic reorganization, subsequent speciation occurred largely without chromosomal rearrangement. The results conflict with the pattern seen in all other species groups of Simuliidae, in which one or more diagnostic rearrangements are typically expressed among species in the same group. The chromosomes provide limited evidence that four entities are valid species but no evidence for the nine others. The weight of evidence from combined chromosomal, molecular, and morphological data, in addition to the practical considerations made for insular species, supports the species status of seven of these nominal species; the remaining taxa require further study. The *S. striatum* group, accordingly, is either over-divided into nominal species or deficient in chromosomal discriminators. If most or all nominal species and unnamed species are valid, all but one are homosequential, an unprecedented condition in Simuliidae. This group illustrates the need for the integration of multiple character sets for discovering and delimiting species.

## Simple Summary

The morphological similarity among black flies often necessitates the use of multiple characters to determine their species status and infer evolutionary relationships. We present a case involving the *Simulium striatum* species group in which chromosomal characters provide almost no distinction among putative species that were originally described based on morphological characters and sometimes molecular data. Although the group is well defined by six unique fixed chromosomal inversions, only 1 of the 13 species we examined had a unique fixed inversion. Based only on chromosomal data, 12 members of the group examined had the same banding pattern, indicating that they are either homosequential species or conspecific. The systematics of the Simuliidae benefit from the opportunity to combine data from multiple character systems. Thus, by combining chromosomal, molecular, and morphological characters, and making practical considerations for insular species, we suggest that at least 7 of the 13 taxa in our study are valid species; more data are needed to determine the status of the other group members studied.

## 1. Introduction

The processes and drivers of speciation have long fascinated biologists [1]. Chromosomal reorganization has been suggested to be one of the mechanisms that initiates the divergence of populations [2,3]. This perspective, however, carries the caveat that species-specific chromosomal rearrangements might be the characteristics of species that appeared after speciation rather than drivers of the process. The role of chromosomes as possible drivers of speciation is perhaps nowhere better documented than in the family Simuliidae, in which structural rearrangements of the complement are abundant across taxa [4,5]. Speciation in the Simuliidae has been associated with chromosomal phenomena, such as translocations, inversions, sex chromosome differentiation, and heterochromatization [5,6,7,8,9]. These evolutionary insights into speciation have been facilitated by the introduction of chromosome band patterns into simuliid taxonomy [10]. Band patterns have also revolutionized our understanding of species and their relationships [11], much like gene sequences have in the past quarter of a century for all organisms [12]. Over the span of about 70 years and with a body of research totaling more than 650 papers, chromosomes have proved their mettle in revealing hundreds of cryptic species and cytological forms of black flies while also revealing monophyletic groups and evolutionary relationships [13]. With few exceptions, chromosomes from the giant larval silk glands provide a unique signature for each species of black fly, regardless of the supraspecific taxon under investigation [4,11]. We present a case in which chromosomal rearrangements play a minimal role in distinguishing species and would have played little to no role in speciation.

The *Simulium striatum* group provides an instructive example of the need for integrated taxonomy [14]. It encourages the recognition that not all character sources are useful in defining species of black flies or their relationships. Early examples of the limitations of character sources in simuliid taxonomy involved the absence of diagnostic morphological characters in all but one or two life stages [15]. The pupal gill is often the only species-level source of discriminators in the *S. striatum* group [16]. Molecular approaches have also had their limitations in distinguishing species in the *S. striatum* group [14].

The *S. striatum* group consists of 43 nominal species distributed in the Oriental Region and at the margins of the Palearctic Region [17,18]. When species groups were first recognized in the Simuliidae, the members of the *S. striatum* group were assigned to the *S. multistriatum* group [19] and later raised to generic status as *Striatosimulium* [20]. Shortly thereafter, *Striatosimulium* was downgraded to its former *S. multistriatum* species group status [21], although the *S. striatum* group had been recognized a decade earlier [22], and was eventually widely embraced [23]. The morphologically supported monophyly of the *S. striatum* group holds up chromosomally and molecularly against the *S. chungi* and *S. multistriatum* groups [24,25,26,27]. Early chromosomal work in Thailand, when only three species in the *S. striatum* group were recognized as existing in the country, did not reveal diagnostic chromosomal rearrangements or DNA sequence differences [14,28].

Here, we elaborate on the need for integrated systematics in the *S. striatum* group by examining the contribution of chromosomal band patterns to species resolution and the role that chromosomes might have played in the speciation of the group.

## 2. Materials and Methods

**Figure 1:**
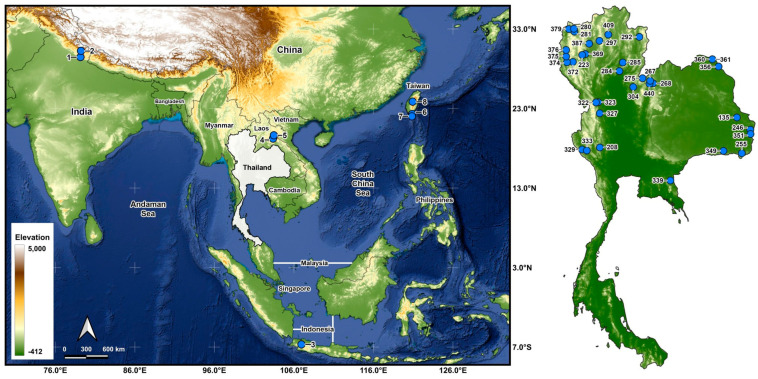
Map of collection sites for the *Simulium striatum* samples used in chromosomal analyses. The numbers beside the blue dots represent the collection sites in Table 1 and Table 2.

Larvae were collected from 43 stream sites in 5 countries (Table 1 and Table 2; Figure 1) and fixed in Carnoy’s fixative (1 part glacial acetic acid: 3 parts 95% ethanol), which was changed 2 or 3 times. Middle to final instars were used for chromosomal analysis. After chromosomal analyses, the larvae were transferred to 80% ethanol for long-term storage and deposited in the Clemson University Arthropod Collection. The parasites in the chromosomally prepared larvae were recorded.

**Figure 2:**
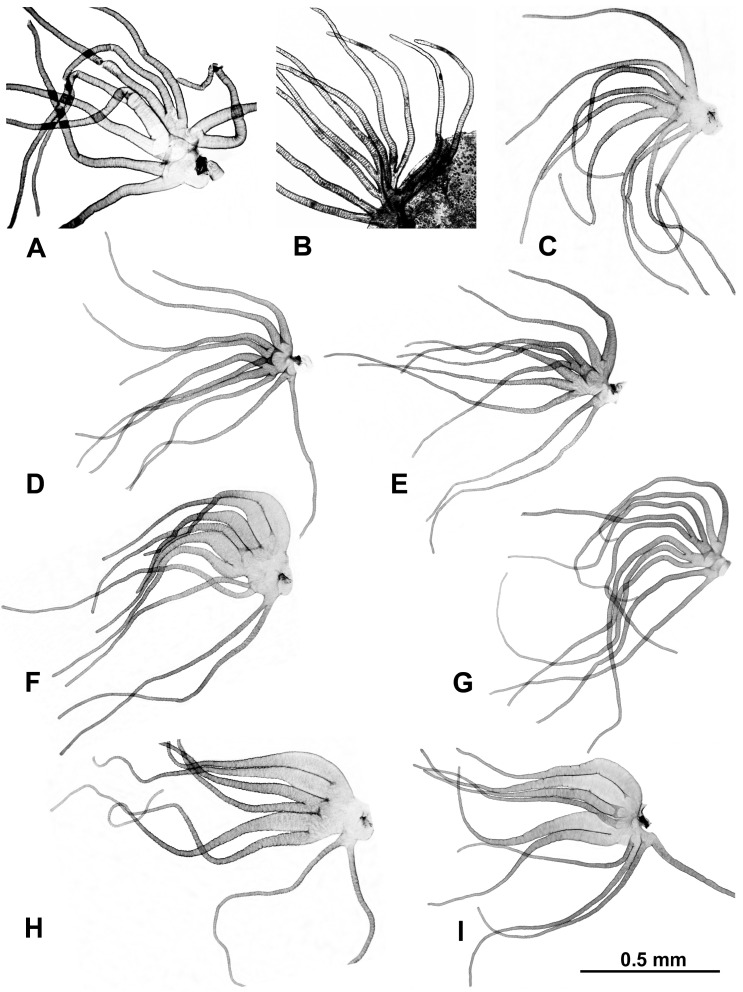
Gills dissected from larvae (unless otherwise indicated); scale bar applies to all gills. (**A**) *Simulium* sp. A from India, site 1. (**B**) *Simulium* sp. B from India (pupal gill), site 2. (**C**) *Simulium maeklongkeense* from Thailand, site 380. (**D**) *Simulium tadtonense* from Thailand, site 292. (**E**) *Simulium* sp. from Laos, site 5. (**F**) *Simulium nakhonense* from Thailand, site 333. (**G**) *Simulium wangkwaiense* from Laos, site 5. (**H**) *Simulium chiangmaiense* from Thailand, 325. (**I**) *Simulium phraense* from Thailand, site 284.

Morphological identifications of species were made using the available keys [16], which were supplemented by original descriptions when more details were needed. Mature (dark) gill histoblasts were dissected from final-instar larvae and placed on a slide in a drop of 50% acetic acid to uncurl. A coverslip was applied with no added pressure, and the preparation was examined under a BH-2 Olympus microscope (Evident Scientific, Waltham, MA, USA). The 2 populations from India, presumably representing 2 different species, based on their gill filament thickness, could not be definitively identified and are referred to as ‘sp. A’ and ‘sp. B’ (Figure 2). One of the 2 species found at site 6 in Laos was identified as *S. tadtonense* Takaoka, Srisuka & Saeung, based on its gills [29] (Figure 2), but the available evidence indicates that it is reproductively isolated from *S. tadtonense*, which occurs at the same site; we therefore refer to the species simply as ‘sp.’.

As for the samples from Thailand, where 10 species of the *S. striatum* group are found [16], we first analyzed their pre-final instars (*n* = 342), including 14 pre-final instars from the type locality of *S. poolpholi* Takaoka, Srisuka & Saeung. We then analyzed their final instars (*n* = 137) by dissecting out a mature gill from each final instar. The configuration of the gill is diagnostic for most species in Thailand [16,29,30,31,32]. When recording chromosomal rearrangements, all fully analyzed larvae (pre-final plus final instars, *n* = 479) were treated *en masse*; in addition, final instars were treated separately according to species, which was determined based on the gill configuration. *Simulium thailandicum* Takaoka & Suzuki, known only from a single male and its associated pupal exuviae, cannot be distinguished from *S. nakhonense* Takaoka & Suzuki based on their gills, nor can *S. thilorsuense* Takaoka, Srisuka & Saeung or *S. concitatum* Srisuka, Takaoka & Saeung be distinguished from *S. wangkwaiense* Takaoka, Srisuka & Saeung by their gills [16,18,31]. We therefore were unable to determine if *S. thailandicum*, *S. concitatum*, and *S. thilorsuense* were present in our larval samples.

The procedures used for Feulgen staining and preparing microscopic slidemounts of chromosomes and gonads (for sex determination) followed standard procedures [33], as did the procedures for the lacto-aceto orcein staining [34]. Band comparisons were made with bright-field microscopy under oil immersion, using *Simulium* subgeneric standard maps [33]. Photographs of the chromosomes were taken with a Jenoptik ProgRes^®^ SpeedXT Core 5 digital camera (Jenoptik, Huntsville, AL, USA) on a BH-2 Olympus microscope. Chromosomal maps were constructed from digital images using Adobe^®^ PhotoShop^®^ Elements 8.

Before comparing all bands in the chromosomal complement from each larva with the subgeneric standard sequence of *Simulium* [33], we screened the diagnostic species group IIS sequence to ensure that only members of the *S. striatum* group were included in our study. This procedure allowed us to exclude 26 larvae (17 females and 9 males) of *S. fenestratum* found in material from Thailand and 3 larvae (1 female and 2 males) from Laos (site 5). The Thailand larvae included 7 larvae homozygous standard for IIIL-2 [8], 16 homozygous inverted for IIIL-2, and 3 polymorphic for IIIL-2, whereas the 3 larvae from Laos were homozygous standard; no other polymorphisms were found in these 29 larvae.

Chromosomal inversions previously found in the *S. chungi*, *S. multistriatum*, and *S. striatum* species groups were numbered according to their original designations [8,27]. New inversions compared to those in the *S. chungi*, *S. multistriatum*, and *S. striatum* groups were given unique numbers according to the arm in which they were discovered. Pericentric inversions were coded using the chromosome and ‘P’ (e.g., IIP-1). Each amplified band (i.e., heteroband, hb) was named according to its chromosome arm and section number (e.g., IIL hb55) as was each large block of heterochromatin (e.g., IS hc18). Secondary nucleolar organizers (2°N.O.) were labelled using their chromosome arm (e.g., IS 2°N.O.). The centromere bands of the 3 chromosomes (CI, CII, and CIII) could occur as a diffuse, rather than sharp, band. Fixed rearrangements within a species are italicized; polymorphisms are not. All rearrangements discovered in our study are indicated, using arrows or brackets, on photomaps of the chromosomes. Section numbers on our chromosome maps follow those of the maps for the *Simulium* subgeneric standard [33].

## 3. Results

### 3.1. Chromosomal Generalities

Identifications based on larval gill histoblasts revealed additional sites for three species previously found in only one location: *S. maeklongkeense* (sites 374, 375, 380, 381, and 387), *S. phraense* (site 284), and *S. tadtonense* (sites 292, 297, 304, 322, 327, and 369). When we found mature larvae of two or more nominal species together in a sample from the same site, their gill configurations (Figure 2) were consistent with those in their original species descriptions, with no intermediate conformations. The following nominal species occurred together: *S. chiangmaiense* and *S. maeklongkeense*, *S. chiangmaiense* and *S. nakhonense*, *S. chiangmaiense* and *S. phraense*, and *S. nakhonense* and *S. tadtonense*.

The banding sequences of 706 (83.2%) of the 849 prepared larvae from 13 taxa of the *S. striatum* group were read entirely; the two samples from India (sites 1 and 2) provided only 44 (36.7%) workable larvae out of 120. Preparations that were not read entirely were excluded from all tabulations and analyses.

**Figure 3:**
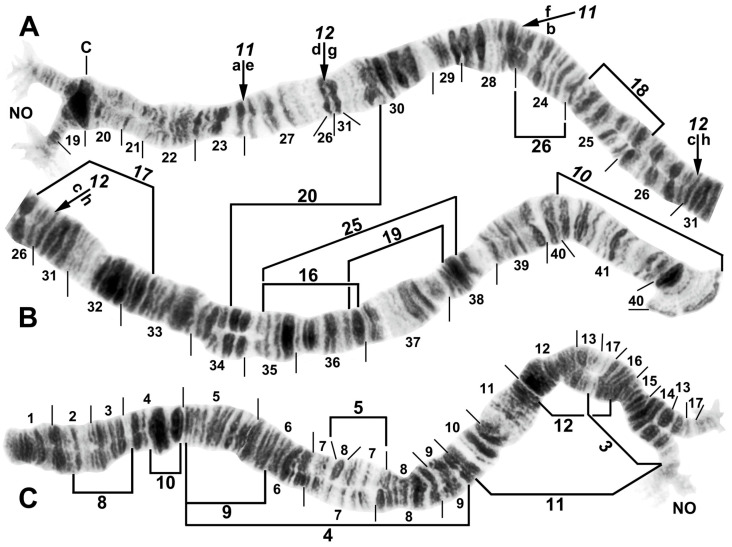
Chromosome I of *Simulium argyrocinctum*, showing the breakpoints of the fixed and floating inversions of the *S. striatum* group; female larvae; C, centromere. (**A**) Proximal half of IL with fixed *IL-11,12* sequence and arrows indicating breakpoints. (**B**) Distal half of IL with fixed *IL-10* sequence. (**C**) IS with fixed *IS-3* sequence and heterozygous configuration of floating inversion IS-5; the upper homologue carries the inverted sequence.

**Figure 4:**
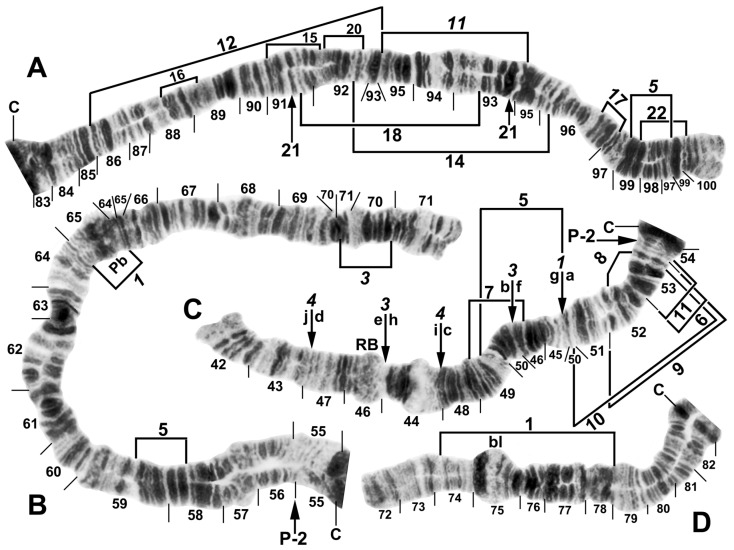
Chromosomes II and III of *Simulium argyrocinctum*, showing the breakpoints of the fixed and floating inversions of the *S. striatum* group. (**A**) IIIL with fixed *IIIL-5*,*11* sequence and brackets indicating inversions; female larva. (**B**) IIL with fixed *IIL-1*,*3* sequence and brackets indicating inversions; female larva. The distal breakpoint of the pericentric inversion IIP-2 is indicated by an arrow at the 55/56 section junction; Pb, parabalbiani. (**C**) IIS with fixed *IIS-1*,*3*,*4* sequence and arrows indicating breakpoints; female larva. Alphabetizing the letters a–j will produce the standard sequence for the subgenus *Simulium*. The proximal breakpoint of the pericentric inversion IIP-2 is indicated by an arrow in section 54; RB, ring of Balbiani. (**D**) IIIS showing standard sequence for the subgenus *Simulium*; male larva; bl, blister.

**Figure 5:**
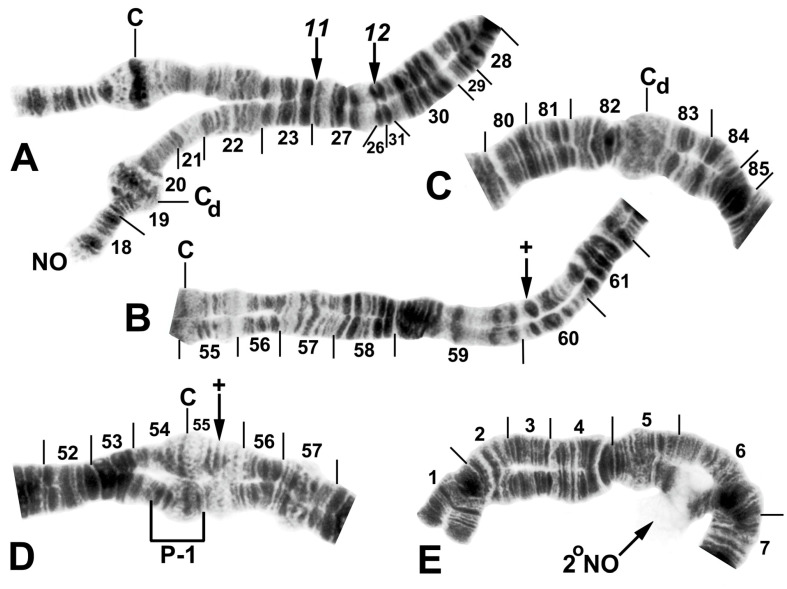
Chromosomes of *Simulium quinquestriatum*. (**A**) Base of IL with proximal breakpoints of *IL-11* and *IL-12* indicated by arrows; female larva; C, sharply defined centromere band; C_d_, diffuse centromere band. (**B**) Base of IIL showing the standard sequence for the subgenus *Simulium* with an arrow indicating the presence of hb60 (+) in the heterozygous condition; female larva; C, centromere. (**C**) Centromere region of III with a homozygous diffuse centromere band (C_d_); female larva. (**D**) Centromere region of II, showing IIP-1 in the heterozygous condition; male larva; arrow indicates presence of hb55′ (+) in the heterozygous condition. (**E**) Distal portion of IS showing heterozygous expression of a secondary nucleolar organizer (2°NO); female larva.

All species had standard arm associations, tightly paired homologues, no chromocenter, and a nucleolar organizer in section 18 in the base of IS (Figure 3A,C). A total of 66 chromosomal rearrangements were found, including fixed inversions (12), floating inversions (40), centromere band dimorphisms (3), heterobands (8), a heterochromatic block (1), a secondary nucleolar organizer (1), and a B (supernumerary) chromosome (1). Of the floating inversions, all but two (5.0%) were paracentric; IIP-1 and IIP-2 were pericentric (Figure 4B,D and Figure 5D).

### 3.2. Species Group Characterization

**Figure 6:**
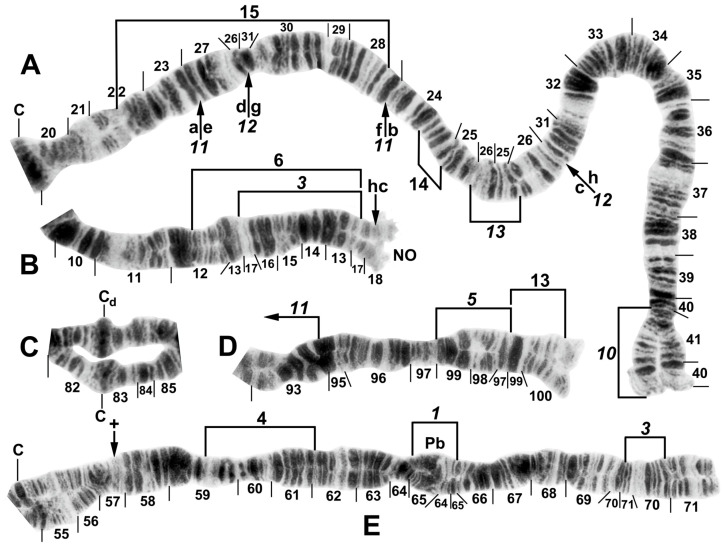
Chromosomes of *Simulium pingtungense*, showing the breakpoints of the fixed and floating inversions of the *S. striatum* group; female larvae. (**A**) IL with fixed *IL-10*,*11*,*12*,*13* sequence and arrows and brackets indicating inversions. Alphabetizing the letters a–h (and inverting *IL-13*) will produce the standard sequence for the subgenus *Simulium* in the proximal half of IL; C, centromere. (**B**) Basal portion of IS with fixed *IS-3* sequence indicated by bracket; NO, nucleolar organizer; location of a heterochromatic block (hc) is indicated by an arrow. (**C**) Centromere region of III. C, sharply defined centromere band; C_d_, diffuse centromere band. (**D**) Distal end of IIIL with fixed *IIIL-5*,*11* sequence. (**E**) IIL with fixed *IIL-1*,*3* sequence and brackets indicating inversions. C, centromere; Pb, parabalbiani; +, location of hb57.

**Figure 7:**
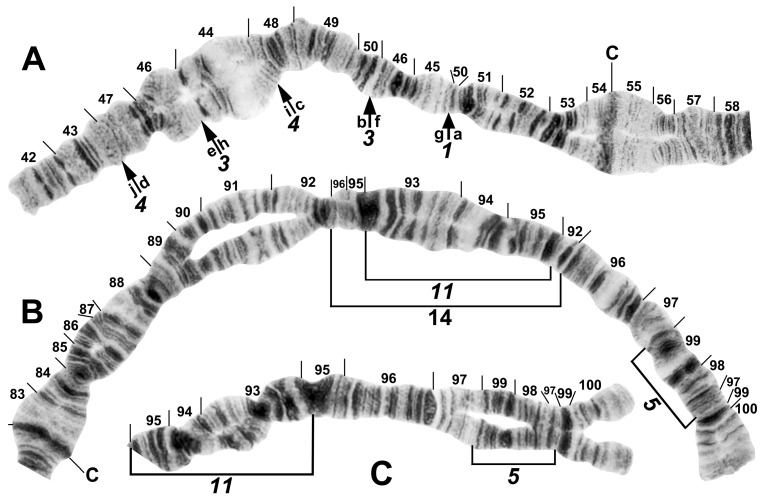
Chromosomes II and III of the *Simulium striatum* group in Laos. (**A**) IIS of *Simulium* sp. with fixed *IIS-1*,*3*,*4* sequence and arrows indicating breakpoints; female larva. Alphabetizing the letters a–j will produce the standard sequence for the subgenus *Simulium*. C, centromere. (**B**) IIIL of *S. tadtonense* with *IIIL-5*,*11*,14 sequence and brackets indicating inversions; C, centromere; male larva. (**C**) Distal half of IIIL of *Simulium* sp. with fixed *IIIL-5*,*11* sequence; male larva.

The basic banding sequence of the *S. striatum* group differed from the *Simulium* subgeneric standard sequence by 11 fixed inversions: *IS-3*, *IL-10*, *IL-11*, *IL-12*, *IIS-1*, *IIS-3*, *IIS-4*, *IIL-1*, *IIL-3*, *IIIL-5*, and *IIIL-11* (Table 3 and Figure 3, Figure 4, Figure 6A,B,D,E and Figure 7A,C). We considered *IS-3* to be fixed, although one male larva from Thailand (site 375) was heterozygous, suggesting a rare retention of the standard sequence.

### 3.3. Interspecific Differences

Only one fixed interspecific rearrangement was found among the 10 nominal species and 3 unidentified species. *Simulium pingtungense* differed from all other species by having *IL-13*, a small inversion easily overlooked as a read-through (Table 3, Figure 6A). Its floating inversions, although low in frequency (≤0.02), were also unique (Figure 6A–E).

**Figure 8:**
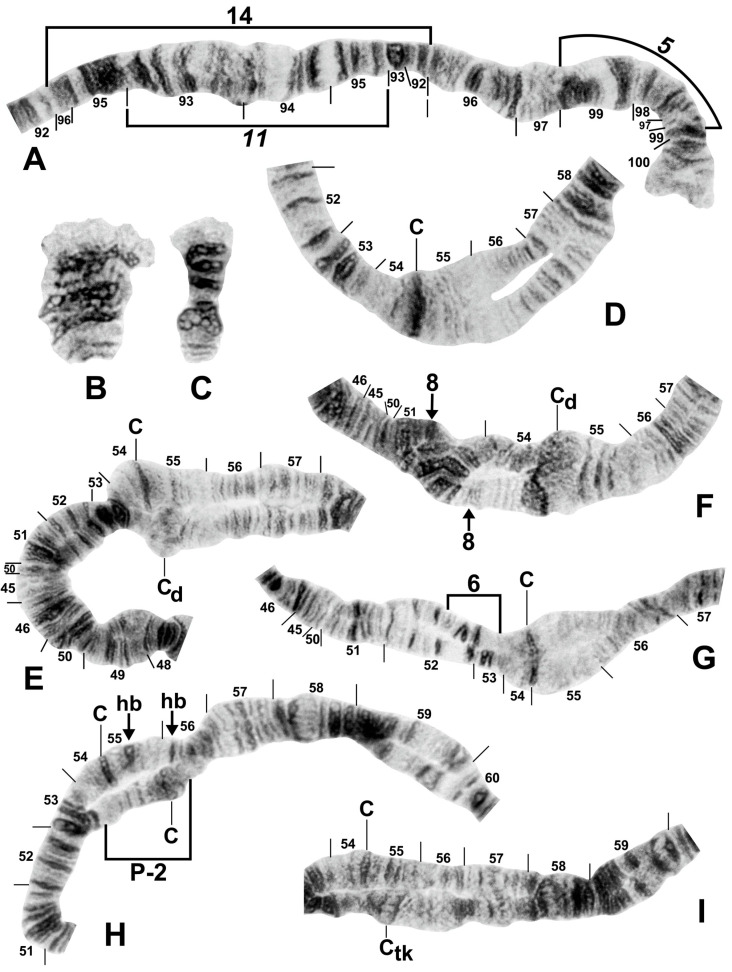
Chromosomes of the *Simulium striatum* group in Thailand. (**A**) IIIL with fixed *IIIL-5*,*11* sequence showing homozygous IIIL-14 condition (brackets); female larva (site 322). (**B**) B chromosomes in *S. wangkwaiense*; female larva (site 440). (**C**) B chromosome of unidentified member of the *S. striatum* group; male larva (site 409). (**D**–**I**) Centromere region of chromosome II; male larvae; C, centromere. (**D**) Typical well-defined homozygous centromere band (arrow), with standard sequences in the base of IIS and IIL (site 349). (**E**) A well-defined centromere band (C) versus a diffuse centromere band (C_d_) and the standard sequence in the base of IIS and IIL (site 292). (**F**) Heterozygous IIS-8 inversion marked with arrows (site 297). (**G**) Heterozygous IIS-6 inversion (bracketed on inverted homologue) (site 329). (**H**) Heterozygous IIP-2 inversion (bracketed on inverted homologue) with heterobands (hb) 55 and 56 marked with arrows (site 339). (**I**) Well-defined centromere band (C) versus thick centromere band (C_tk_) with the standard sequence in the base of IIL (site 323).

**Figure 9:**
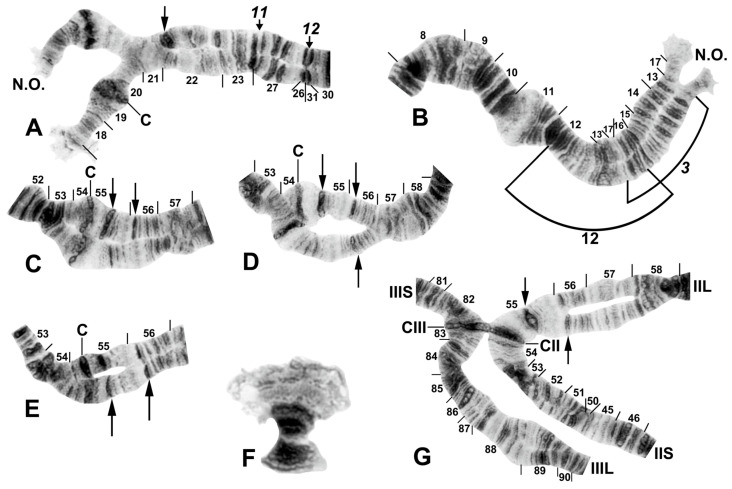
Chromosomes of *Simulium* sp. from Laos. (**A**) Base of IL, with nucleolar organizer (N.O.) in base of IS; male larva. Breakpoints of fixed inversions *IL-11* and *IL-12* are shown with short arrows; longer arrow shows heterozygous expression of heteroband IL hb22. (**B**) Base of IS with *IS-3* sequence and the limits of floating inversion IS-12 bracketed; N.O., nucleolar organizer; male larva. (**C**–**E**) Centromere region of chromosome II; C, centromere; female larvae. (**C**) Heterozygous heterobands IIL hb55 and hb56 are shown with arrows. (**D**) Heterozygous heterobands IIL hb55, hb56 (top homologue), and hb56′ (lower homologue) are shown with arrows. (**E**) Homozygous heterobands IIL hb55 and hb56 are shown with arrows. (**F**) B chromosomes; female larva. (**G**) Ectopic pairing of centromere bands CII and CIII in male larva; homozygous heterobands IIL hb55 and hb56 are highlighted using arrows.

One inversion, IIIL-14, was restricted to *S. tadtonense* (Figure 8A). Although not fixed, it occurred predominantly (65.4%) in the homozygous condition (4 ss, 5 si, and 17 ii, where s = standard and i = inverted) and was exclusively associated with this species (Table 4). The most telling examples of its status in signalling reproductive isolation were found at site 327 in Thailand, where only IIIL-14 homozygotes (23) and standard homozygotes (4) were found, corresponding to *S. tadtonense* and *S. chiangmaiense*, respectively, and, although based on a small sample, at site 5 in Laos, where one IIIL-14 homozygote (Figure 7B) occurred in a population of 76 larvae, with the remainder having the standard sequence (Figure 7C). The occurrence of one IIIL-14 homozygote without any heterozygotes is improbable in a panmictic population unless it represents a separate species (*S. tadtonense*) from *S.* sp. The high frequency (≥0.45) of the heterobands IIL hb55 and IIL hb56 (Figure 9) in *S.* sp. at site 5 in Laos also supported the possibility of a unique population, and possibly a cryptic species. Floating inversions (Figure 4A,D and Figure 9A,B), although rare, were unique to *S.* sp.

### 3.4. Intraspecific Variation

**Figure 10:**
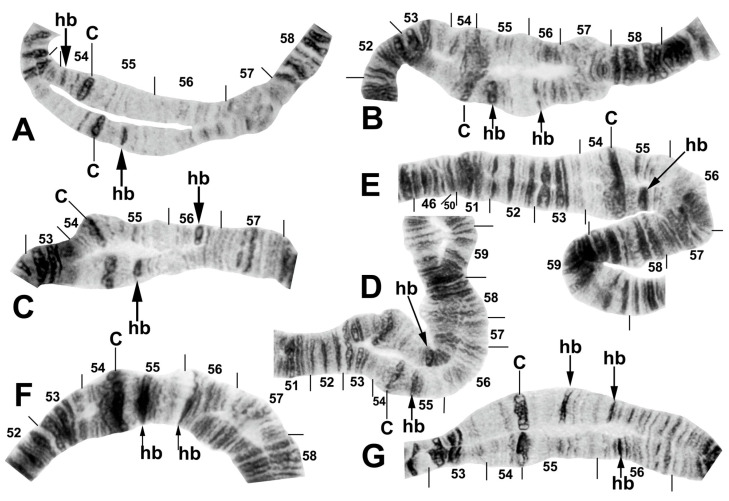
Centromere region of chromosome II in members of the *Simulium striatum* group in Thailand. C, centromere; hb, heteroband. (**A**) Arrows indicate heterozygous heterobands IIS hb54 and IIL hb55 (site 255); female larva. (**B**) Arrows indicate heterozygous heterobands 55 and 56 (site 339); male larva. (**C**,**D**) Arrows indicate heterozygous heterobands 55 and 56′ (sites 297 and 339, respectively); male larvae. (**E**) The arrow indicates heterozygous heteroband 55 (site 284); female larva. (**F**) The arrows indicate homozygous heteroband 55 and heterozygous heteroband 56 (site 369); female larva. (**G**) The arrows indicate heterozygous heterobands 55, 56, and 56′ (site 369); male larva.

Among all larvae, 54 floating rearrangements were discovered and mapped (Table 3, Figure 3, Figure 4, Figure 5, Figure 6, Figure 7, Figure 8, Figure 9 and Figure 10). Half were single-homologue occurrences and another 10 (19.2%) were found in only two homologues. Centromere dimorphisms occurred in all three chromosomes and were expressed as a diffuse band, in contrast to the typical sharply defined, well-stained band commonly seen (Figure 5A,C, Figure 6C and Figure 8E,F), or as a thick, rather than thin, band (Figure 8I). A single secondary nucleolar organizer was found in IS (Figure 5E). All floating rearrangements occurred at frequencies of less than 19%, except for IIL hb55 in *S.* sp. and IIIL-14 in *S. tadtonense* (Table 3). The ectopic pairing of centromeres, most often involving CII and CIII (Figure 9G), was rare in all populations except at site 5 in Laos, where it ranged from 0 to 70% of nuclei, with a mode of 10%.

In Thailand, 10 (40.0%) of the 25 floating rearrangements found were associated with a particular species, as was the presence of B chromosomes in 1 of 2 larvae (Table 4). Some rearrangements found in a particular species (Table 4) might have been present in other species but could not be assigned because they were not found in mature larvae with associated gill histoblasts available for species identification.

The centromere region of chromosome II was particularly active, with centromere band dimorphisms and pericentric inversions in addition to heterobands and paracentric inversions on both sides of the centromere (Figure 4B,C, Figure 5B,D, Figure 8E–I, Figure 9C–E and Figure 10A–G). Heterobands were expressed especially frequently in the IIL arm. Only one heteroband (IL hb22) was found outside of chromosome II (Figure 9A). The most common heteroband was IIL hb55, which was found in one population in Laos and six nominal species in Thailand (Table 3 and Table 4), suggesting a shared ancestry. The heteroband was thickened and darkly stained (Figure 10B), in contrast to the thin or diffuse, often lightly stained, band of the standard sequence (Figure 8D). Among the nominal species in Laos and Thailand, only *S. wangkwaiense* lacked heterobands. When two heterobands occurred in the centromere region of chromosome II, they tended to be in cis or trans configuration. Hb55 and hb56 (the first band in section 56) could occur independently but were typically linked (Figure 8H, Figure 9C–E and Figure 10B,F,G), whereas hb55 and hb56′ (the third band in section 56) occurred on opposite homologues in Laos and Thailand (Figure 9D and Figure 10C,D,G). In Laos (site 5), for example, hb55 and hb56 occurred together on the same homologue 78.6% of the time and never on opposite homologues. In Laos (site 5), hb55 and hb56 were both in Hardy–Weinberg equilibrium for *S.* sp., suggesting a panmictic population. Although IIS hb54 and IIL hb55 could occur independently, they were on opposite homologues in 8 of 9 cases when they occurred in the same larva (Figure 10A).

No rearrangement was sex-linked. Thus, the sex chromosomes were cytologically undifferentiated (X_0_Y_0_) among all 13 taxa.

B chromosomes were found in only three larvae: one from Laos (site 5) and two from Thailand (sites 409 and 440). The banding patterns of the B chromosomes in larvae from Thailand (Figure 8B,C) differed from those in Laos (Figure 9F).

## 4. Discussion

The chromosomal band patterns demonstrate the cohesive nature of the *S. striatum* species group, which was first established and further corroborated morphologically [16,22,35] and supported by molecular analyses [25,27]. Six unique inversions support the group’s monophyly [27], which are named and mapped here for the first time as *IS-3*, *IL-10*, *IL-11*, *IL-12*, *IIS-4*, and *IIIL-11*. We note one caveat, however, when claiming monophyly based on DNA and chromosomes. Although the monophyly of the *S. striatum* group stands up to related species groups that have been evaluated chromosomally and molecularly, particularly the *S. chungi* and *S. multistriatum* groups [27], other morphologically similar groups that were once part of the *S. griseifrons* group [35] have not been adequately studied chromosomally or molecularly.

Several chromosomal features of the *S. striatum* group conform to the trends seen in other species groups. First, the discovery of 2 pericentric inversions (5.0%) among the 40 floating inversions found is low, as is the percentage seen across 65 species [36]; the overwhelming majority were paracentric inversions. Second, the absence of cytological sex-chromosome differentiation between females and males is part of a trend seen in related groups of Southeast Asian black flies, such as the *S. chungi* and *S. multistriatum* groups [8,27]. This pattern might be widespread among tropical black flies more generally, compared to groups in temperate regions, and would be worth evaluating quantitatively. Third, the low frequency (0.4%) of B chromosomes in the *S. striatum* group is comparable to that in other species groups of Southeast Asian black flies [8,37,38], except one high-elevation (>1400 m above sea level) species [39]. The maintenance of B chromosomes in some low-elevation species at minimal frequencies suggests that when environmental conditions merit it, the Bs could be mustered to provide additional variability.

At odds with the trend seen in all other simuliid taxa [13], however, is the near complete lack of chromosomal differentiation within the *S. striatum* group, especially given that at least 13 putative species from two zoogeographic regions (Oriental and Palearctic), on the mainland and on islands (e.g., Java and Taiwan), were evaluated, with linear distances between populations of more than 4000 km. This group represents a singular example of chromosomes failing to provide diagnostic discriminators for most of the nominal species in a taxon. A previous study failed to find separation along four axes (chromosomal, ecological, molecular, or morphological) for two nominal species, *S. chiangmaiense* and *S. nakhonense*, suggesting synonymy [14], and the primary morphological discriminator, 8 versus 10 gill filaments, respectively, was shown to be variable [40]. A molecular study using the big zinc finger (*BZF*) gene [30], however, indicated that *S. chiangmaiense* and *S. nakhonense* are distinct species. A later molecular study, also using *BZF*, indicates that *S. chiangmaiense* and *S. nakhonense* are distinct, whereas the *COI* barcoding and elongator complex protein 1 (*ECP1*) genes fail to differentiate them [25].

The plethora of nominal species in the *S. striatum* group in Thailand—nine described species, of which we analyzed seven chromosomally—is an example of small morphological differences among species and a failure of certain genes (e.g., *COI*) to distinguish the species, while other genes (*BZF*) can be used to identify them [25,31]. Molecular analyses of some of these species, however, are based on small samples from single locations, raising the risk of location effects, particularly when geographic distances between similar nominal species are considerable. Adding to the confusion is the finding that two species in our study, *S. nakhonense* and *S. wangkwaiense*, might consist of six species each, based on molecular analyses [25].

Further adding to the complexity is a population of *S.* sp. in Laos that features larvae with a gill configuration identical to that of *S. tadtonense* but lacking the floating inversion IIIL-14, as well as one larva homozygous for IIIL-14 (unique to *S. tadtonense*). The occurrence of one larva homozygous for IIIL-14 among all others at the site provides limited evidence that *S.* sp. is a cryptic species. The case is strengthened by the high frequency of heterobands in the base of IIL in *S.* sp. The possible existence of a cryptic species of *S. tadtonense* raises the question of which entity is the true *S. tadtonense*. In the absence of chromosomal information from the type locality of *S. tadtonense*, we arbitrarily regard the presence of IIIL-14 as a mark of true *S. tadtonense* and its absence as a mark of *S.* sp. In contradistinction, the lack of heterobands, especially the common IIL hb55, in *S. wangkwaiense* in Laos and Thailand might have some diagnostic chromosomal value at a population level; all other examined nominal species in these countries have heterobands on one or both sides of CII.

The chromosomal data suggest that either the *S. striatum* group is over-split into nominal species, several of which are found in only a single location, or that chromosomes fail to distinguish legitimate species. The chromosomes provide marginal to moderate support that four segregates in our study are valid species (*S. pingtungense*, *S. tadtonense*, *S. wangkwaiense*, and *S.* sp.) but no chromosomal support for the nine others (*S. argyrocinctum*, *S. chiangmaiense*, *S. maeklongkeense*, *S. nakhonense*, *S. phraense*, *S. poolpholi*, *S. quinquestriatum*, *S.* sp. A, and *S.* sp. B). Accordingly, the determination of species status requires an integrated approach. We found several species, previously identified based only on their respective type localities, at additional sites and with consistent gill structure. Combining chromosomal, molecular, and new morphological data (i.e., the gill consistency of nominal species at sites beyond the type locality), we view the following nominal species in our study as valid: *S. maeklongkeense*, *S. nakhonense*, *S. phraense*, *S. pingtungense*, *S. quinquestriatum*, and *S. tadtonense*. This conclusion is not to say that other entities in our study are not valid species, but only that additional data would be desirable. Our study provides no new evidence for or against the species status of *S. chiangmaiense* independent of *S. nakhonense*. Although our results suggest that *S. wangkwaiense* in Laos and Thailand is a valid species, further study of its distinction from *S. quinquestriatum* is warranted; more than 2000 km separate their type localities. The nominal species *S. concitatum*, *S. thilorsuense*, and *S. thailandicum*, which have been found at single sites in Thailand, have gill configurations like those of other species in our study and are defined on the basis of minute morphological differences and, for the first two, the *BZF* gene. If the larvae of any of these three species were present in our study, they were not chromosomally or morphologically detectable.

Determining species status is particularly problematic for insular populations isolated from mainland areas by more than 100 km and must be evaluated in the context of strict allopatry. *Simulium argyrocinctum*, for example, is one such species in this situation. In such cases, the weight of evidence from different data sources, typically morphological, molecular, and chromosomal, can be used to reach a rational decision about species status [41]. The morphological differences of *S. argyrocinctum* are subtle [42] and its chromosomal differences are nonexistent. However, the *COI* barcode gene indicates that *S. argyrocinctum* is part of an operational taxonomic unit (with *S. baliense* Takaoka & Sofian-Azirun) distinct from the eight analyzed mainland nominal group members that, themselves, are not distinct from one another [27]. Thus, the weight of evidence regarding the species status of *S. argyrocinctum* is ambivalent. From a purely pragmatic perspective, we continue to recognize *S. argyrocinctum* as a valid species, with its primary diagnostic feature being its apparent restriction to Java and Sumatra, as the only member of the *S. striatum* group on these islands, although conceivably it might also occur in Malaysia, where two other nominal species of the *S. striatum* group are found. Another insular species, *S. pingtungense*, is the only analyzed member of the group that expresses a fixed chromosomal difference. Given the allopatric nature of the Taiwan population of *S. pingtungense*, this fixed difference cannot by itself be considered indicative of a unique biological species (i.e., reproductively isolated), except in comparison with another chromosomally analyzed species from Taiwan, *S. quinquestriatum*; both species have their type localities in Taiwan, and the populations of the two species we analyzed were about 190 km apart.

The influence of environment on many structural characters is not known, obscuring the differences between site-specific and species differences. Experiments have shown that the number of primary rays in the labral fan of larvae changes with current speed and food availability [43,44]. Anecdotal evidence suggests that other characters, such as the amount of silk in the cocoon and the number of hooklets in the posterior proleg, also are subject to environmental influence [45,46]. The effect of the environment on pupal characters, such as the branching pattern and surface sculpture of the gills, which are used in the diagnosis of members of the *S. striatum* group, is unknown for simuliids in general. Molecular differences, as with morphological differences, in some cases might reflect adaptation to local ecological conditions, especially if dispersal is restricted and females tend to return to their natal streams. Dispersal distances for members of the *S. striatum* group are unknown but worthy of investigation.

Taxonomy has implications for assessing practical issues such as public health and habitat conservation. Some members of the *S. striatum* group are vectors of onchocercid parasites that can cause zoonotic onchocerciasis [47], and an accurate identification of these vectors is required. Although black flies as organisms are rarely considered for conservation [48], they are excellent indicators of unique aquatic habitats. Cases such as that illustrated by the *S. striatum* group pose a quandary for biologists. The apparent geographic restriction of some nominal species in the group suggests that their aquatic habitats and surrounding environments are unique, but the question remains as to whether populations or reproductively isolated species are being evaluated.

Overall, the separation of the *S. striatum* group from all other known species groups in the Simuliidae, based on six fixed inversions, suggests that chromosomes could have played a role in the origin of the group. However, in the group’s subsequent evolutionary history, chromosomal rearrangements were only minimally, if at all, involved in speciation. Thus, nearly all evaluated species in the group are homosequential, while bordering on being homosequential cryptic species [49], that is, species that share the same morphology and chromosomal banding sequence. The differences among nominal species are minimal when all three data sets (chromosomal, molecular, and morphological) are considered, suggesting the possibility that some nominal species represent a single species. On the other hand, their enormous geographic distribution across multiple ecoregions suggests that at least some of the chromosomally nondifferentiated populations represent valid species. Few nominal species that have been screened as cryptic species have such vast distributions [50]. Thus, the dilemma stands: are the members of the *S. striatum* group an exception to the pattern that species in the Simuliidae are chromosomally differentiated, obviating the possibility that chromosomal rearrangements played a role in the group’s speciation, or do they conform to the pattern but have been divided too finely into putative species? Although some nominal species in the group stand definitively as biological species, a robust integrated approach involving larger samples from more locations is needed to fully resolve the species-level taxonomy of all members of the group.

## Figures and Tables

**Table 1:** Collection sites for larvae of the *Simulium striatum* group.

Site	Location	Longitude Latitude	Elevation (Masl)	Collection Date	Species (*n*)
1	India, Uttarakhand, Nainital District, Cataliakot River (2.5–3.5 m wide), 900 m above mouth	29°28′25″ N 79°09′39″ E	456	2 May 2018	sp. A (33)
2	India, Uttarakhand, Chamoli District, Pindar River (15–20 m wide), 1 km above Karan-prayg	30°15′05″ N 79°13′24″ E	785	6 May 2018	sp. B (11)
3	Indonesia, Java, Cugur Cilember	06°39′39″ S 106°56′46″ E	884	25 July 2018	*argyrocinctum* (39)
4	Laos, Xiangkhouang Province, Muang Khoun	19°13′41″ N 103°21′57″ E	990	8 February 2025	*wangkwaiense* (19)
5	Laos, Xiangkhouang Province, Muang Kham	19°38′16″ N 103°28′26″ E	750	9 February 2025	sp. (75), *tadtonense* (1)
6 ^1^	Taiwan, Pingtung County, Manjhou, Jioupeng	22°06′41″ N 120°52′28″ E	50	3 February 2008	*pingtungense* (23)
7 ^1^	Taiwan, Pingtung County, Manjhou, Lide	22°01′02″ N 120°50′25″ E	50	3 February 2008	*pingtungense* (21)
8 ^2^	Taiwan, Nantou Province, Yuchi Township	23°52′ N 120°56′ E	670	1 March 2006	*quinquestriatum* (6)
–	Thailand, 35 sites (Table 2)	(Table 2)	(Table 2)	(Table 2)	*striatum* group (479)

^1^ Same site, including the type locality (Jioupeng), as that from which material was collected for the description of *S. pingtungense*; ^2^ same site as that from which material was collected for the redescription of *S. quinquestriatum*.

**Table 2:** Collection sites for larvae of the *Simulium striatum* group in Thailand.

Site	Location	Longitude Latitude	Elevation (masl)	Collection Date	Larvae Analyzed (*n*)
135	Amnat Chareon Province, Huai Nang Koi waterfall, Phathum Ratchawongsa	15°53′32″ N 104°54′20″ E	160	23 February 2008	3
208	Kanchanaburi Province, Tham Than Lod, Si Sawat	14°40′02″ N 99°19′09″ E	276	30 September 2008	7
223	Chiang Mai Province, Mae Ya waterfall, Chomthong	18°26′23″ N 98°35′53″ E	587	21 November 2008	9
246 ^1^	Ubon Ratchathani Province, Huai Phang	15°23′28″ N 105°27′16″ E	189	1 November 2009	14
255	Ubon Ratchathani Province, Kang Lam Duan waterfall, Nam Khun	14°26′05″ N 105°06′22″ E	179	2 November 2009	14
267	Loei Province, Pla Ba waterfall, Phuruea	17°23′19″ N 101°22′11″ E	649	24 November 2009	7
268	Loei Province, Song Khon waterfall, Phuruea	17°21′08″ N 101°24′14″ E	743	26 November 2009	16
275 ^2^	Loei Province, Thansawan waterfall, Na Haeo	17°29′27″ N 101°03′34″ E	506	27 November 2009	10
284	Uttaradit Province, Huai Cham Phang	17°47′00″ N 100°06′56″ E	130	5 December 2009	11
285	Uttaradit Province, Ban Mae Kam	18°08′01″ N 100°15′20″ E	236	5 December 2009	12
292	Nan Province, Ban Rong Ngae, Pua	19°10′31″ N 100°55′49″ E	257	6 December 2009	19
297 ^2^	Chiang Mai Province, Mae Klang waterfall, Chomthong	19°00′59″ N 99°18′11″ E	615	6 December 2009	25
304	Phitsanulok Province, Chat Trakan waterfall	17°07′56″ N 100°40′52″ E	194	8 December 2009	3
322	Kamphaeng Phet Province, Klong Wang Chao waterfall,	16°30′16″ N 99°09′36″ E	236	19 February 2010	31
323 ^3,4^	Kamphaeng Phet Province, Klong Wang Chao	16°30′15″ N 99°10′03″ E	204	19 February 2010	27
327	Kamphaeng Phet Province, Klong Khlung, Klong Lan	16°03′43″ N 99°18′53″ E	118	20 February 2010	27
329	Kanchanaburi Province, Ban Huai Khayeng	14°35′06″ N 98°35′29″ E	237	20 February 2010	11
333	Kanchanaburi Province, Huai U-long	14°32′45″ N 98°47′13″ E	172	20 February 2010	16
339	Chanthaburi Province, Khao Soi Dao waterfall	13°19′18″ N 102°11′38″ E	316	3 March 2010	10
349 ^2^	Sisaket Province, Huai Chan waterfall, Khun Han	14°31′48″ N 104°21′32″ E	116	15 October 2010	10
351	Ubon Ratchathani Province, Sirindhorn	15°12′56″ N 105°27′46″ E	114	16 October 2010	11
356	Nakhon Phanom Province, Tad Kham waterfall, Ban Phaeng	17°58′08″ N 104°09′34″ E	122	17 October 2010	10
360	Bueng Kan Province, Jed Si waterfall (1), Se ka	18°15′51″ N 103°54′21″ E	169	18 October 2010	9
361 ^2,4^	Bueng Kan Province, Jed Si waterfall (2), Se ka	18°15′51″ N 103°54′21″ E	169	18 October 2010	12
369 ^2,5^	Chiang Mai Province, Mae Klang waterfall, Chomthong	18°29′39″ N 98°40′07″ E	304	16 January 2015	11
372 ^2^	Chiang Mai Province, Huai Mae Um long	18°09′22″ N 98°13′06″ E	852	16 January 2015	10
374	Mae Hong Son Province, Mae Sariang	18°08′33″ N 97°58′35″ E	203	16 January 2015	12
375	Mae Hong Son Province, Mae La Noi	18°22′41″ N 97°56′32″ E	270	16 January 2015	12
376	Mae Hong Son Province, Khun Yuam	18°38′39″ N 97°56′29″ E	471	16 January 2015	16
379	Mae Hong Son Province, Mueang Mae Hong Son	19°29′45″ N 98°03′21″ E	495	17 January 2015	24
380 ^6^	Mae Hong Son Province, Pang Mapha	19°30′37″ N 98°15′46″ E	610	17 January 2015	15
381	Mae Hong Son Province, Ban Pang Paek, Pang Mapha	19°26′14″ N 98°15′49″ E	787	17 January 2015	12
387 ^7^	Chiang Mai Province, Mae Rim	18°54′02″ N 98°52′55″ E	508	18 January 2015	16
409 ^2^	Lampang Province, Wang Thong waterfall	19°16′16″ N 99°39′50″ E	510	18 December 2015	8
440	Loei Province, Song Khon waterfall, Phuruea	17°21′14″ N 101°25′20″ E	721	27 May 2017	19

^1^ Type locality of *S. poolpholi*. ^2^ A subset of the larvae from each of these sites and dates was molecularly identified as *S. wangkwaiense* [25]. ^3^ A subset of the larvae from this site and date was molecularly identified as *S. chiangmaiense* [25]. ^4^ A subset of the larvae from each of these sites and dates was molecularly identified as *S. nakhonense* [25]. ^5^ About 1 km from the type locality of *S. wangkwaiense*. ^6^ A subset of the larvae from this site and date, termed “unknown larvae” [25], was molecularly identified as *S. nakhonense* (*n* = 2), *S. phraense* (*n* = 1), and *S. tadtonense* (*n* = 1). ^7^ A subset of the larvae from this site and date, termed “unknown larvae” [25], was molecularly identified as *S. nakhonense* (*n* = 4), and *S. tadtonense* (*n* = 1).

**Table 3:** Frequency of rearrangements in the larvae of members of the *Simulium striatum* group.

Taxon ^1^	argyr.	ping.	quin.	tad.	wang.	sp.	sp. A	sp. B	*striatum* Group
Site	3	6, 7	8	5	4	5	1	2	9
Country	Indon. (Java)	Taiw.	Taiw.	Laos	Laos	Laos	India	India	Thailand
F:M	23 ^2^:16	26 ^3^:18	4:2	0:1	12:7	38:37 ^4^	12:20:1 ^5^	4:7	249:230 ^6^
*IS-3*	1.00	1.00	1.00	1.00	1.00	1.00	1.00	1.00	0.999
IS-4	0.10								
IS-5	0.01								
IS-6		0.02							
IS-8								0.18	
IS-9								0.14	
IS-10									0.001
IS-11							0.02		
IS-12						0.007			
IS hc18 ^7^		0.01							
IS 2°NO			0.08						
*IL-10*	1.00	1.00	1.00	1.00	1.00	1.00	1.00	1.00	1.00
*IL-11*	1.00	1.00	1.00	1.00	1.00	1.00	1.00	1.00	1.00
*IL-12*	1.00	1.00	1.00	1.00	1.00	1.00	1.00	1.00	1.00
*IL-13*		1.00							
IL-14		0.01							
IL-15		0.01							
IL-16							0.18		0.001
IL-17							0.02		
IL-18								0.18	
IL-19								0.09	
IL-20									0.001
IL-25									0.001
IL-26									0.001
IL hb22						0.02			
CI diffuse			0.08						
*IIS-1*	1.00	1.00	1.00	1.00	1.00	1.00	1.00	1.00	1.00
*IIS-3*	1.00	1.00	1.00	1.00	1.00	1.00	1.00	1.00	1.00
*IIS-4*	1.00	1.00	1.00	1.00	1.00	1.00	1.00	1.00	1.00
IIS-5									0.001
IIS-6									0.002
IIS-7									0.001
IIS-8									0.002
IIS-9									0.003
IIS-10									0.002
IIS-11									0.001
IIS-basal ^8^									0.001
IIS hb54									0.01
*IIL-1*	1.00	1.00	1.00	1.00	1.00	1.00	1.00	1.00	1.00
*IIL-3*	1.00	1.00	1.00	1.00	1.00	1.00	1.00	1.00	1.00
IIL-4		0.01							
IIL-5									0.001
IIL hb55						0.57 ^9^			0.17
IIL hb55′			0.08						
IIL hb56						0.45 ^9^			0.02
IIL hb56′						0.08			0.003
IIL hb57		0.01							
IIL hb60			0.08						
CII diffuse									0.003
IIP-1			0.08						
IIP-2									0.002
IIIS-1						0.007			
*IIIL-5*	1.00	1.00	1.00	1.00	1.00	1.00	1.00	1.00	1.00
*IIIL-11*	1.00	1.00	1.00	1.00	1.00	1.00	1.00	1.00	1.00
IIIL-12	0.01								
IIIL-13		0.01							
IIIL-14				1.00					0.11
IIIL-15									0.001
IIIL-16									0.001
IIIL-17							0.03		
IIIL-18								0.14	
IIIL-20									0.002
IIIL-21									0.002
IIIL-22						0.01			
CIII diffuse		0.02							
Bs (%) ^10^	0.0	0.0	0.0	0.0	0.0	1.3	0.0	0.0	0.004

Note: Open cells indicate a frequency of 0. ^1^ *argyr.*, *S. argyrocinctum* De Meijere; *ping.*, *S. pingtungense* Huang & Takaoka; *quin*., *S. quinquestriatum* (Shiraki); *tad*., *S. tadtonense* Takaoka, Srisuka & Saeung; *wang*., *S. wangkwaiense* Takaoka, Srisuka & Saeung. ^2^ One female was infected with a protozoan resembling *Tetrahymena.* ^3^ One female was infected with a microsporidian resembling *Polydispyrenia simulii*. ^4^ Larvae (7 females and 4 males) from Laos were infected with small, slender mermithid nematodes, much thinner than those infecting larvae in Thailand. ^5^ Sex could not be determined for 1 larva. ^6^ Larvae from the following sites in Thailand (Table 2) were infected with unidentified mermithid nematodes: sites 223 (5 females), 325 (1 female), 369 (2 females), 372 (1 female), 376 (1 female and 1 male), and 380 (2 females). One female larva at site 374 was infected with the fungus *Coeolomycidium simulii*. ^7^ IS 18 hc = heterochromatic block in section 18; the block was on the same homologue as IS-6 in one male. ^8^ Basal heterozygous inversion with breakpoints unresolved. ^9^ The following heterobands were in Hardy–Weinberg equilibrium (*p* > 0.05, df = 1): IIL hb55 (−− = 17, +− = 31, ++ = 27, where − = standard band and + = amplified band; χ^2^ = 1.8811); IIL 56 hb (−−= 24, +− = 35, ++ = 16; χ^2^ = 0.2346). ^10^ B chromosomes were present in 1 female larva in Laos and 1 female and 1 male larva in Thailand.

**Table 4:** Frequency of rearrangements and percentage of B chromosomes (Bs) in final-instar larvae, or in a population of larvae at a type locality (for *S. poolpholi* only), of members of the *Simulium striatum* group in Thailand.

Species ^1^	*chiang.*	*maek.*	*nakhon.*	*phra.*	*poolph.*	*tadton.*	*wangk.*
F:M	16:13	11:9	5:6	0:1	3:11	13:12	23:28
IS-8		0.02					
IL-20							0.01
IIS-9		0.02					
IIS-10	0.02						
IIS-11	0.02						
IIS hb54					0.07		
IIL hb55	0.24	0.15	0.14	0.50	0.29	0.30	
IIL hb56		0.10	0.09			0.04	
IIL hb56′			0.05				
IIIL-14						0.74	
IIIL-20					0.04		
Bs (%)							1.96

^1^ *chiang.*, *S. chiangmaiense*: sites 284 (0:1), 285 (1:2), 323 * (0:1), 325 (2:3), 375 (1:0), 376 (3:1), and 379 (9:5). * A subset of larvae from this site and collection date was molecularly identified as *S. chiangmaiense* [25]. *maek.*, *S. maeklongkeense*: sites 374 (2:1), 375 (1:0), 380 (4:1), 381 (2:1), and 387 (2:6). *nakhon.*, *S. nakhonense*: sites 285 (1:0), 297 (0:3), 329 (1:0), and 333 (3:3). *phra.*, *S. phraense*: site 284 (0:1). *poolph.*, *S. poolpholi*: site 246 (type locality) (3:11). *tadton.*, *S. tadtonense*: sites 292 (4:2), 297 (2:4), 304 (1:0), 322 (5:5), 327 (0:1), and 369 (1:0). *wangk.*, *S. wangkwaiense*: sites 223 (8:1), 255 (2:1), 268 (2:5), 275 * (3:7), 351 (1:0), 360 (4:5), 361 * (0:2), 409 * (0:1), and 440 (3:6). * A subset of larvae from each of these sites and collection dates was molecularly identified as *S. wangkwaiense* [25].

## Data Availability

The data supporting the reported results are contained in this paper. Further inquiries can be directed to the corresponding author.
